# The age-related contribution of cognitive function to dual-task gait in middle-aged adults in Spain: observations from a population-based study

**DOI:** 10.1016/S2666-7568(23)00009-0

**Published:** 2023-03

**Authors:** Junhong Zhou, Gabriele Cattaneo, Wanting Yu, On-Yee Lo, Natalia A Gouskova, Selma Delgado-Gallén, Maria Redondo-Camós, Goretti España-Irla, Javier Solana-Sánchez, Josep M Tormos, Lewis A Lipsitz, David Bartrés-Faz, Alvaro Pascual-Leone, Brad Manor

**Affiliations:** Hinda and Arthur Marcus Institute for Aging Research (J Zhou PhD, W Yu ME, O-Y Lo PhD, N A Gouskova PhD, Prof L A Lipsitz MD, Prof A Pascual-Leone MD, B Manor PhD) and Deanna and Sidney Wolk Center for Memory Health (Prof A Pascual-Leone), Hebrew SeniorLife, Roslindale, MA, USA; Department of Neurology (Prof A Pascual-Leone) and Division of Gerontology, Beth Israel Deaconess Medical Center (J Zhou, O-Y Lo, N A Gouskova, Prof L A Lipsitz, B Manor), Harvard Medical School, Boston, MA, USA; Institut Guttmann, Institut Universitari de Neurorehabilitació adscrit a la UAB, Barcelona, Spain (G Cattaneo PhD, S Delgado-Gallén MSc, M Redondo-Camós MSc, G España-Irla MSc, J Solana-Sánchez PhD, J M Tormos MD, Prof D Bartrés-Faz PhD); Department of Medicine, Universitat Autònoma de Barcelona, Barcelona, Spain (G Cattaneo, S Delgado-Gallén, M Redondo-Camós, G España-Irla, J Solana-Sánchez, J M Tormos); Department of Medicine, Faculty of Medicine and Health Sciences, Institute of Neurosciences and August Pi i Sunyer Biomedical Research Institute, University of Barcelona, Barcelona, Spain (Prof D Bartrés-Faz)

## Abstract

**Background:**

Poor dual-task gait performance is associated with a risk of falls and cognitive decline in adults aged 65 years or older. When and why dual-task gait performance begins to deteriorate is unknown. This study aimed to characterise the relationships between age, dual-task gait, and cognitive function in middle age (ie, aged 40–64 years).

**Methods:**

We conducted a secondary analysis of data from community-dwelling adults aged 40–64 years that took part in the Barcelona Brain Health Initiative (BBHI) study, an ongoing longitudinal cohort study in Barcelona, Spain. Participants were eligible for inclusion if they were able to walk independently without assistance and had completed assessments of both gait and cognition at the time of analysis and ineligble if they could not understand the study protocol, had any clinically diagnosed neurological or psychiatric diseases, were cognitively impaired, or had lower-extremity pain, osteoarthritis, or rheumatoid arthritis that could cause abnormal gait. Stride time and stride time variability were measured under single-task (ie, walking only) and dual-task (ie, walking while performing serial subtractions) conditions. Dual-task cost (DTC; the percentage increase in the gait outcomes from single-task to dual-task conditions) to each gait outcome was calculated and used as the primary measure in analyses. Global cognitive function and composite scores of five cognitive domains were derived from neuropsychological testing. We used locally estimated scatterplot smoothing to characterise the relationship between age and dual-task gait, and structural equation modelling to establish whether cognitive function mediated the association between observed biological age and dual tasks.

**Findings:**

996 people were recruited to the BBHI study between May 5, 2018, and July 7, 2020, of which 640 participants completed gait and cognitive assessments during this time (mean 24 days [SD 34] between first and second visit) and were included in our analysis (342 men and 298 women). Non-linear associations were observed between age and dual-task performance. Starting at 54 years, the DTC to stride time (β=0·27 [95% CI 0·11 to 0·36]; p<0·0001) and stride time variability (0·24 [0·08 to 0·32]; p=0·0006) increased with advancing age. In individuals aged 54 years or older, decreased global cognitive function correlated with increased DTC to stride time (β=−0·27 [−0·38 to −0·11]; p=0·0006) and increased DTC to stride time variability (β=−0·19 [−0·28 to −0·08]; p=0·0002).

**Interpretation:**

Dual-task gait performance begins to deteriorate in the sixth decade of life and, after this point, interindividual variance in cognition explains a substantial portion of dual-task performance.

## Introduction

Walking is often performed simultaneously with other cognitive tasks, such as talking, reading signs, or making decisions. The regulation of gait, especially under such dual-task conditions, relies on numerous cognitive functions.^1^ In adults aged 65 years or older, even subtle cognitive impairments are associated with unsteadiness (ie, a high degree of temporospatial variability over consecutive strides), increased dual-task costs (DTCs; ie, gait disturbances induced by performing a concurrent cognitive task, which is often obtained by calculating the percent change in the gait performance from single tasks to dual tasks), and increased risk of falls.^2,3^ Diminished dual-task gait performance at baseline is also predictive of future cognitive decline.^4,5^ Montero-Odasso and colleagues reported that older adults with mild cognitive impairment who had relatively high DTC to gait speed were more likely to develop dementia in the next 2 years.^4^ However, a causal relationship has not been established, and increased DTC might be one of the earliest symptoms of an underlying neurodegenerative disease that has not yet led to dementia. In any case, the observed associations between gait and cognitive function have resulted in an increased emphasis on the measurement of dual-task gait as a means of assessing cognitive function and brain health and their effects on daily life activities.^6^

Research on the interconnectedness of gait and cognition has focused on differences between healthy younger (ie, aged <40 years) and older (ie, aged ≥65 years) adults, differences between groups of older adults with dissimilar cognitive function, and changes in function over time in older adults.^7,8^ However, little is known regarding the role of cognitive function in the control of gait during middle age (ie, aged 40–64 years), nor when or how this association changes during this period. Middle age is often the life period during which age-related diseases, including most neurodegenerative diseases (eg, dementia), first manifest as measurable functional decline.^9^ We propose that the associations between gait, age, and cognitive function in middle age are important to understand because early identification of functional decline and subsequent intervention are likely to be crucial for the preservation of functional independence in old age.

We aimed to characterise the cross-sectional associations between age, dual-task gait performance, and cognitive function in middle age. We completed a secondary analysis of data from the Barcelona Brain Health Initiative (BBHI) study, an ongoing longitudinal cohort study aimed at identifying the determinants of brain health maintenance in middle-aged adults without overt neurological or psychiatric conditions.^10^ Our primary hypotheses were that dual-task gait performance, as quantified by the DTC to stride time and stride time variability, would diminish with individual age within this middle-aged cohort and that the observed associations between DTC to gait and age would be mediated by global cognitive function.

## Methods

### Study design and participants

We did a secondary analysis of data from participants in the BBHI study, Barcelona, Spain. All participants were aged 40–65 years, community-dwelling, and able to walk without assistance. Exclusion criteria were an inability to understand the study protocol, any overt, clinically diagnosed neurological (eg, Parkinson’s disease or peripheral neuropathy) or psychiatric (eg, depression) disorder, cognitive impairment as defined by a Mini Mental State Examination score of 24 or less,^11^ and lower-extremity pain, osteoarthritis, or rheumatoid arthritis that could cause abnormal gait. Participants were recruited with various methods, including advertisements in newspapers and social media, press releases on television, and posters in the local community or companies. People who were interested in participating were contacted via telephone or the project website. Potential participants completed remote and in-person screening to participate in the BBHI study. Sex was self-reported via multiple choice: “man”, “woman”, or “other (please specify)”. All the study protocols involving human participants were approved by the ethics committee of Unió Catalana d’Hospitals (approval number CEI 18/07). All participants provided written informed consent to participate. The BBHI study protocol has previously been published elsewhere.^10^

### Procedures

Each participant completed detailed clinical phenotyping (appendix pp 1–2) at the Guttmann Brain Health Institute, Barcelona, Spain.^10^ We used data from participants who had completed both gait and cognitive assessments at the time of this analysis.

Staff-supervised gait assessments were completed using a validated smartphone app (appendix p 1).^12,13^ Each assessment comprised one 45 s trial of quiet walking at preferred speed and one 45 s trial of walking at preferred speed while performing verbalised serial subtractions of 3 from a random three-digit number.^4^ Gait measurements were derived from the motion data acquired by the smartphone app (with periods of turning omitted) and included the mean stride time (ie, the time between two consecutive heel strikes of one foot) and stride time variability (ie, the coefficient of variation about the mean stride time) for each trial.^12,13^ Designated primary gait measures included the DTC to stride time and the DTC to stride time variability (ie, the percent change of stride time and stride time variability from single-task to dual-task conditions).^4,12^ Exploratory gait measures were stride time and stride time variability within single-task and dual-task conditions separately. Serial subtraction performance (ie, percentage of correct responses) was also calculated for each dual-task trial.

In a separate visit, participants completed a neuropsychological assessment led by a clinical neuro-psychologist. The assessment battery included well established tests that together assess several different cognitive domains.^14^ A global cognitive function composite score was constructed from performance in each neuropsychological test.^14^ Composite scores of five latent cognitive factors pertaining to processing speed, working memory, episodic memory, flexibility, and reasoning were also calculated (appendix pp 1–2). Notably, composite score construction was computed using the entire BBHI cohort and, as such, the scores of the subset included in the present analysis were not necessarily zero-mean (appendix pp 5–6); in other words, individual positive (ie, better than the mean) and negative (ie, worse than the mean) values reflect performance variance in relation to the entire BBHI cohort.

### Outcomes

The primary outcome was the association between age and DTC to stride time and DTC to stride time variability across the entire study cohort. Secondary outcomes included the association between global cognitive score and DTC to stride time and DTC to stride time variability, the contribution of specific cognitive domains to gait performance within each age group (ie, younger and older group), and whether cognitive function mediated the association between gait performance and age within the older group.

### Statistical analysis

The normality of data distribution (including the residuals in regression analysis) was examined with the Shapiro–Wilk test and the homogeneity of variance was examined with Levene’s test. The linearity between variables in regression analyses was also examined by using the plot of residual and fitted value. The potential confounders of the regression and structural equation model analyses were identified from previous literature and incorporated into a causal directed acyclic graph to guide the modelling strategy (appendix p 3).^15^ Significance level was set at p<0·025 using Bonferroni’s correction for multiple comparisons for the two primary gait measures (ie, DTC to stride time and DTC to stride time variability).

First, we examined the association between each primary gait measure and age with a multistep approach. We began by visually examining the nature of these associations using locally estimated scatterplot smoothing (LOESS) plots.^16^ We established that the association between gait and age followed a piecewise linear curve and defined a potential age threshold at which the association between age and gait changes. Specifically, several regression models were fitted, each using a different value for age as thresholds (ie, the visually identified changepoint plus or minus 3 years), and the age threshold with the highest *R*^*2*^ was selected. On the basis of the age threshold from our primary analysis, we categorised all participants into younger (ie, 42–53 years) and older (ie, 54–64 years) groups. To compare demographic characteristics, gait, and cognitive performance between groups, we used one-way ANOVA models when the data were normally distributed with homogeneity of variance or the Kruskal-Wallis test when the data were not normally distributed. We used χ^2^ analysis to examine the difference in sex between groups.

Second, we used linear regression models to examine the association between the primary gait measures and age within younger and older groups. Sex and its interaction with age were included in the model.

Third, we used linear regression models to characterise the association between global cognitive score and each primary gait measure. Age group (ie, younger or older) and its interaction with cognitive function was included in each model. We also characterised the contribution of specific cognitive domains (ie, working memory, processing speed, episodic memory, flexibility, and reasoning) to gait performance within each age group by use of multivariable regression analyses that simultaneously included composite scores of these domains. Age, sex, and BMI were used as the covariates.

Fourth, we examined whether cognitive function mediated the association between gait performance and age by use of a structural equation model with robust maximum likelihood estimation. Because no association between age and gait performance was observed in the younger group, we limited the mediation analysis to the older group only. The DTC to stride time and stride time variability were included as the outcome measures in the structural equation model and the global cognitive score was included as the mediator. The standardised coefficients of each individual path were tested for significance to show the relative magnitude of the association on each path, and the natural associations (eg, direct and indirect association) were examined. The p value for the χ^2^ statistic, root mean square error of approximation, comparative fit index, and Tucker-Lewis index were used as model indices to assess model fit. A p value of more than 0·05 for the χ^2^ statistic, a root mean square error of approximation of less than 0·06, and a comparative fit index and Tucker-Lewis index of 0·95 or higher were considered indicative of a good model fit. Sex and BMI were included as covariates. A bootstrapping method was used to calculate the 95% bias corrected and accelerated confidence interval (95% BCa CIs) around the mediated and direct associations.

Similar linear regression models and multivariable regression analyses were used in exploratory analyses to examine the associations between exploratory gait measures (ie, the gait outcomes within single-task and dual-task conditions), cognitive function (including specific cognitive domains), and age.

Statistical analyses were performed with JMP Pro 14 (SAS Institute, Cary, NC, USA) and Mplus 8.4 (Muthén and Muthén, Los Angeles, CA, USA).

### Role of the funding source

The funders of the study had no role in study design, data collection, data analysis, data interpretation, or writing of the report.

## Results

Of the 4206 participants initially enrolled in the BBHI study, 996 participants completed in-person assessments between May 5, 2018, and July 7, 2020. The mean time between first and second visit was 24 days (SD 34). We included data from 640 participants (298 [46·6%] women and 342 [53·4%] men) aged 42–64 years who had completed both gait and cognitive assessments at the time of our analysis.

No significant differences were observed in age, BMI, education, Mini Mental State Examination score, or proportions of each sex between the full BBHI cohort and the subset included in this analysis. Participant characteristics are shown in table 1. All 640 participants included in this analysis had high global cognitive function (ie, Mini Mental State Examination total score of 27–30).

Notably, interparticipant variation existed in specific cognitive tests (appendix pp 5–6) and in serial subtraction performance within the dual-task gait paradigm. The mean interval between gait and neuropsychological assessments was 24 days (SD 34).

**Figure 1:**
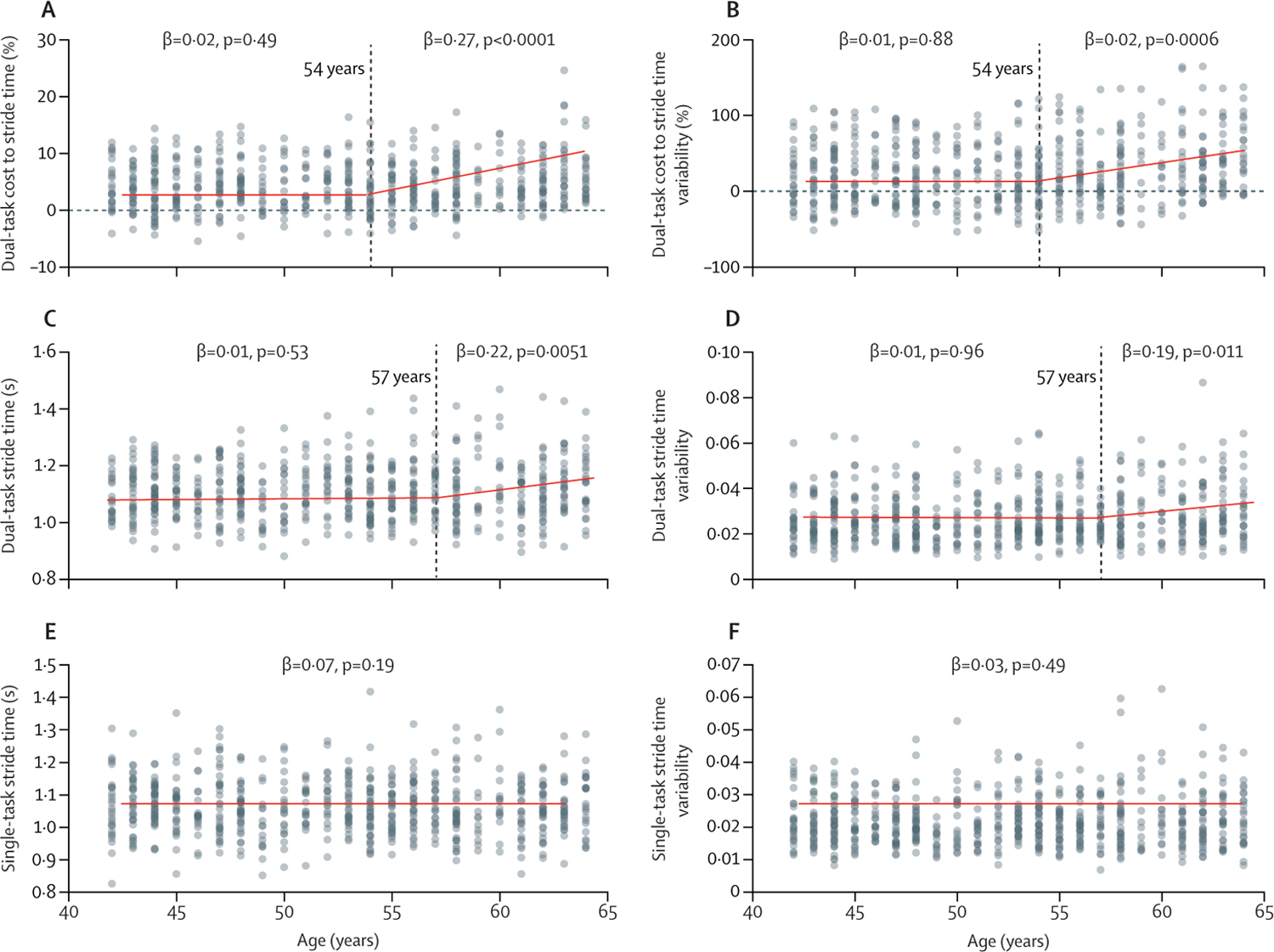
Associations between age and gait performance in single-task and dual-task conditions The dual-task cost to stride time (A) and stride time variability (B) were significantly associated with age after the threshold of 54 years. Within the older group (≥54 years), advancing age was associated with greater (worse) dual-task costs. Within the dual-task condition, mean stride time (C) and stride time variability (D) were significantly associated with age after the threshold of 57 years. Again, within the older group (≥57 years), advancing age was associated with greater (worse) stride time and stride time variability. No such associations were observed in the younger group or between single-task gait performance and age. Vertical dashed lines indicate the age threshold.

LOESS scatter plots showed piecewise linear relationships between age and dual-task performance. Starting at approximately age 54 years, the DTC to stride time and stride time variability started to progressively increase with advancing age (figure 1A, B). A series of regression models, fitted with an age threshold ranging from 51 years to 57 years, indicated that, for both DTC metrics, a threshold of 54 years provided the highest *R*^2^ (DTC to stride time *R*^2^=3%; DTC to stride time variability *R*^2^=3%) of the fitted LOESS curve. Using this threshold of 54 years, we categorised the sample into younger (ie, 42–53 years) and older (ie, 54–64 years) groups. The results of the between-group comparisons in gait and cognitive performance are shown in table 1. Regression models confirmed LOESS results. Compared with the younger group, the correlates between age and DTC to stride time (p=0·012) and DTC to stride time variability (p=0·0031) were significantly greater in the older group. In the older group, age was correlated with DTC to stride time (β=0·27 [95% CI 0·11 to 0·36]; p<0·0001; figure 1A) and stride time variability (β=0·24 [0·08 to 0·32]; p=0·0006; figure 1B), and no significant associations of sex (p=0·21 for DTC to stride time; p=0·37 for DTC to stride time variability) and its interaction with age (p=0·33 for DTC to stride time; p=0·17 for DTC to stride time variability) to DTCs were observed. No such association was observed in the younger group (DTC to stride time: β=0·02 [95% CI −0·03 to 0·04]; p=0·49; DTC to stride time variability β=0·01 [−0·12 to 0·10]; p=0·88).

**Figure 2:**
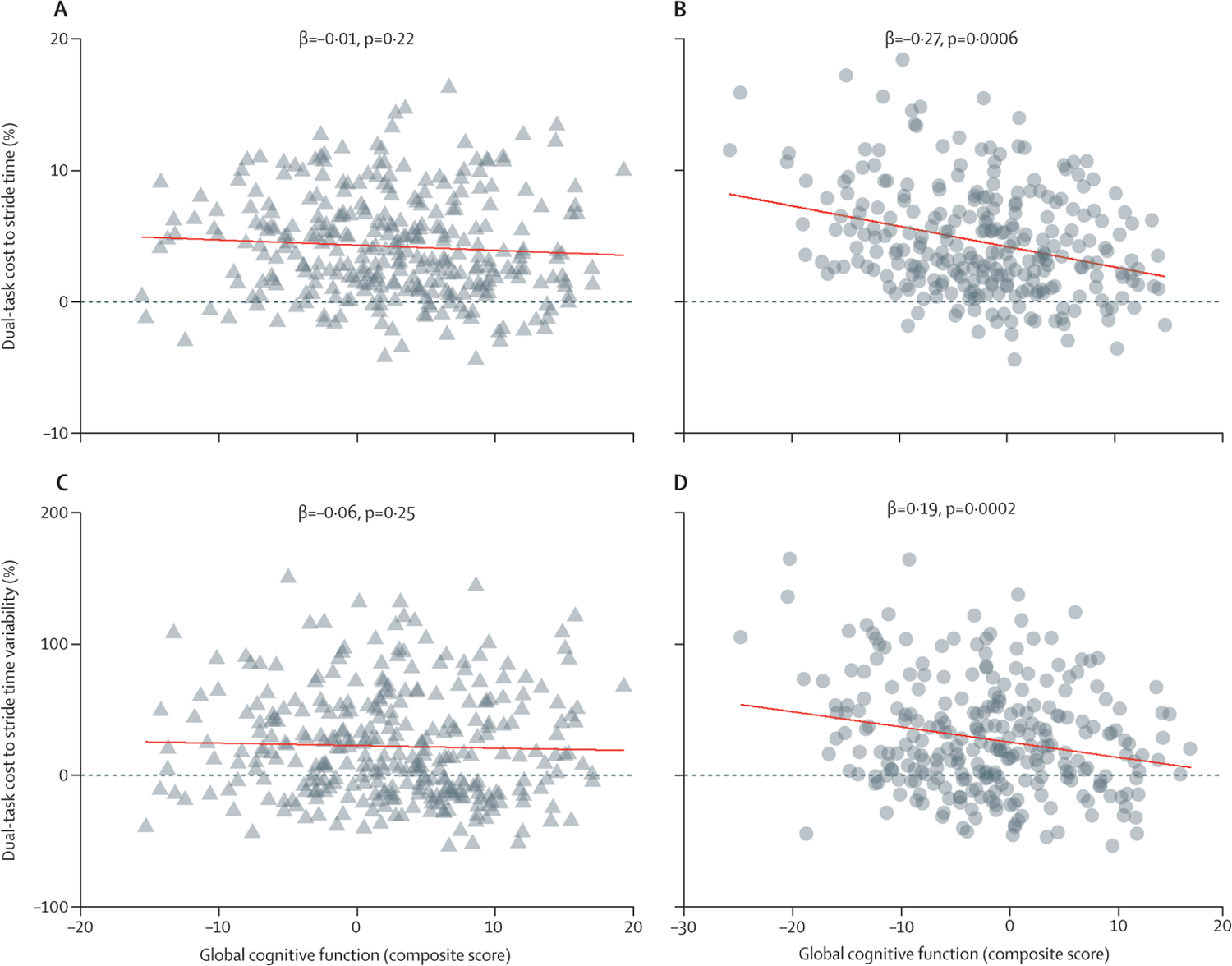
Associations between global cognitive function and dual-task cost to stride time and stride time variability within the younger and older groups Correlations between dual-task cost to stride time and global cognitive function as a composite score in the younger group (ie, aged <54 years; A) and the older group (ie, aged ≥54 years; B). Correlations between dual-task cost to stride time variability in the younger group (C) and the older group (D).

An interaction between age group and global cognitive function for DTC to stride time (p=0·021) and stride time variability (p=0·015) was observed. This interaction indicated that the association between cognitive function and the DTC to each gait metric was different between the younger and older groups. Although global cognitive function was not correlated with DTC to stride time (β=−0·10 [95% CI −0·13 to 0·02]; p=0·22; figure 2A) or stride time variability (β=−0·06 [−0·9 to 0·06]; p=0·25; figure 2C) in the younger group, it was correlated with both DTC to stride time (β=−0·27 [−0·38 to −0·11]; p=0·0006; figure 2B) and stride time variability (β=−0·19 [−0·28 to −0·08]; p=0·0002; figure 2D) in the older group. Multivariable regression analyses showed that, within the older group, processing and working memory were the only two cognitive domains in which performance was correlated with the DTCs to stride time and stride time variability (table 2). Performance in the domains of flexibility, episodic memory, and reasoning was not significantly associated with either gait metric. No significant associations were observed in the younger group or between the covariates and gait outcomes (appendix pp 7–8).

**Figure 3:**
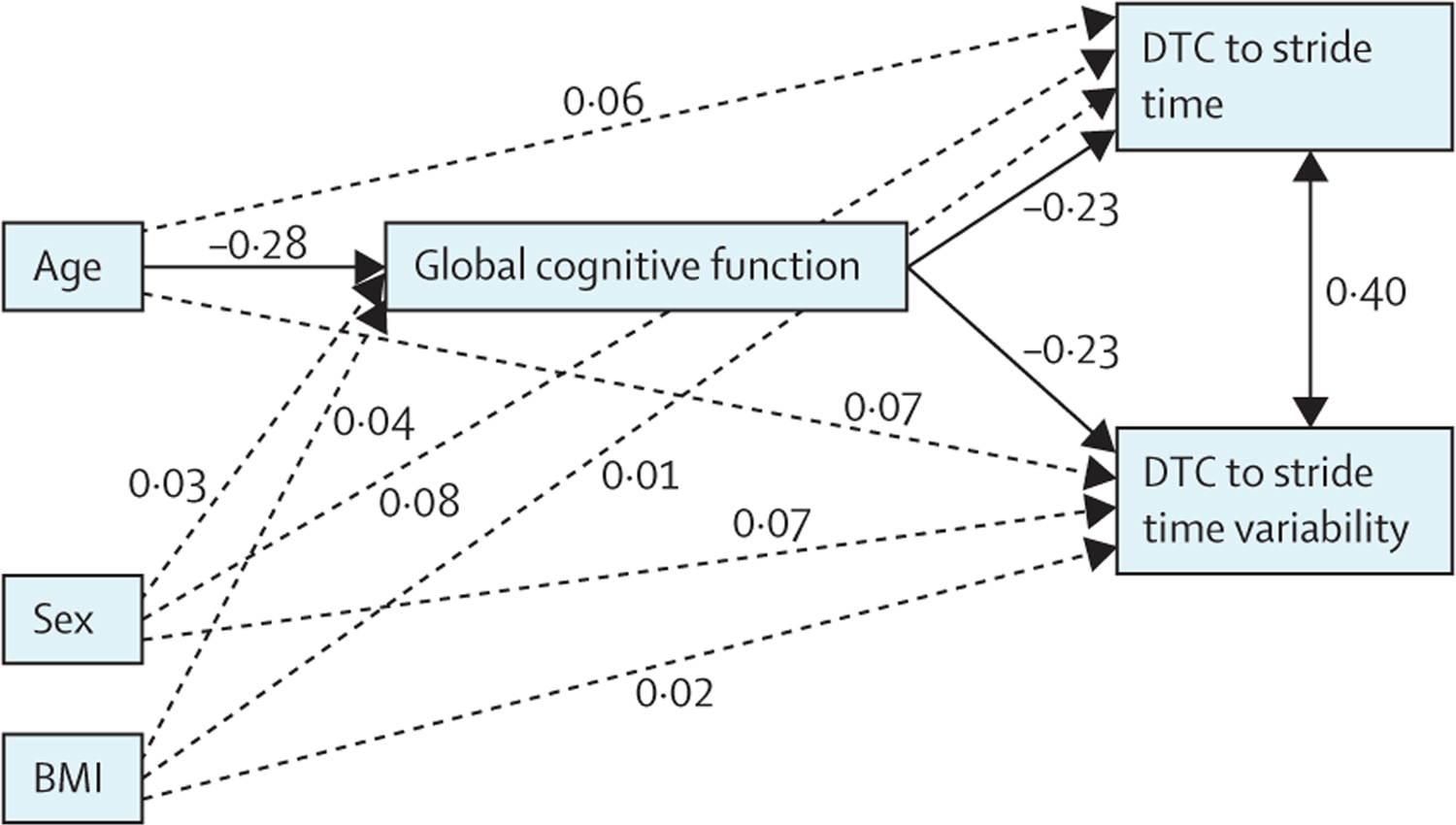
Structural equation modelling of the associations between age, gait, and global cognitive function Values are standardised coefficients. Mediation analysis was completed only within the older group (ie, aged ≥54 years) because no significant associations between age and gait were observed within the younger group (ie, aged <54 years). Significant associations are depicted with solid lines and non-significant associations are depicted with dashed lines. DTC=dual-task cost.

Structural equation model analyses conducted in the older group (ie, aged 54–64 years) showed that global cognitive function mediated the association between age and DTCs to stride time and stride time variability (figure 3). The models showed an excellent model fit (χ^2^=0·80, df=2, p=0·65 for DTC to stride time; χ^2^=0·85, df 2, p=0·78 for DTC to stride time variability; root mean square error of approximation=0·01, comparative fit index=0·98, and Tucker-Lewis index=0·97 for both of the gait outcomes). With global cognitive function included in the model, there was no significant direct association of age on DTC to either gait outcome (DTC to stride time p=0·11; DTC to stride time variability p=0·23). Age, however, was directly associated with global cognitive function (standardised β=−0·28, p<0·0001), and global cognitive function was in turn directly associated with DTC to stride time (standardised β=−0·23; p=0·0012) and stride time variability (standardised β=−0·23; p=0·0012). The total association of age on DTC to stride time was 0·15 (p=0·012), with an indirect association via global cognitive function of 0·06 (p=0·0042). Using these values, we calculated that global cognitive function mediated 43% (95% BCa CI 0·04–0·28) of the association between age and DTC to stride time (standardised coefficient between age and global cognitive function × standardised coefficient between cognitive function and gait outcome / total association between age and DTC to stride time). Similarly, the total association of age on DTC to stride time variability was 0·14 (p=0·031), with an indirect association via global cognitive function of 0·07 (p=0·0052), indicating that cognitive function mediated 47% (95% BCa CI 0·05–0·31) of such association between age and DTC to stride time variability, calculated by use of the formula described previously.

In the exploratory analyses, the LOESS and regression models showed that single-task stride time and stride time variability were not significantly associated with age before or after any specific threshold (figure 1E–F). In the exploratory analyses, LOESS analysis indicated that the optimal age threshold beyond which gait metrics derived from the dual-task condition began to worsen with advancing age was 57 years (dual-task stride time *R*^2^=2·8%; dual-task stride time variability *R*^2^=2·5%; figure 1C, D). Regression models showed that, within the older group (ie, ≥57 years, as determined by dual-task gait performance), participants with older age had greater dual-task stride time (β=0·22 [95% CI 0·03 to 0·24]; p=0·0051; figure 1C) and dual-task stride time variability (β=0·19 [0·08 to 0·28]; p=0·011; figure 1D), and no significant influences of sex (p=0·28 for stride time; p=0·33 for stride time variability) and its interaction with age (p=0·88 for stride time; p=0·19 for stride time variability) on dual-task stride time or dual-task stride time variability were observed. No such associations were observed in the younger group (dual-task stride time β=0·01 [95% CI −0·03 to 0·05]; p=0·53; dual-task stride time variability β=0·01 [−0·03 to 0·06]; p=0·96).

In the exploratory analysis, within the older group (ie, aged ≥57 years, as determined by dual-task gait performance), participants with better global cognitive function had lower dual-task stride time (β=−0·16 [95% CI −0·30 to −0·10]; p=0·014) and stride time variability (β=−0·17 [−0·29 to −0·08]; p=0·0034). Processing speed and working memory were the only domains that were correlated with dual-task stride time variability (table 2). No such associations were observed within the younger group or between any other cognitive domains and dual-task stride time. No significant associations were observed between cognitive outcomes and single-task stride time variability or between the covariates and gait outcomes (appendix pp 7–8).

## Discussion

In this analysis of data from the BBHI cohort, we observed that the DTCs to gait performance, and gait performance specifically within the dual-task condition, decreased linearly with advancing age after the ages of 54 years (for DTCs to gait performance alone) and 57 years (for gait performance within the dual-task condition). In the subsets of the cohort composed of participants older than these ages, the association between age and dual-task gait performance was mediated by global cognitive function. These observations suggest that the ability to maintain gait performance when performing two tasks begins to decline up to a decade before the typically defined threshold for older adults (ie, 65 years) and that, after the age threshold of 54 years, individual differences in cognitive function account for more than 43% of such age-related decline.

Dual-task performance depends largely on the availability of cognitive resources, the capacity of the brain to effectively allocate these resources to each task, and the speed at which the brain can process information related to the execution of each task.^17,18^ Evidence suggests that ageing alters each of these factors and thus leads to greater DTC to performance in one or both involved tasks.^17,18^ Our observations of individuals without neurological or psychiatric disease suggest that dual-task capacity—in this case, specifically, the capacity to maintain walking performance while verbalising mental arithmetic—begins to diminish during the sixth decade of life. The age threshold of DTC to gait performance was 3 years earlier than that of the gait performance within the dual-task condition. This difference might be due to the possibility that, by adjusting for the locomotor elements in gait regulation (which are the same in both single-task and dual-task walking conditions), the cost metric more directly reflects the functionality of the higher-level brain functions that enable dual-task performance (eg, task switching), which might be particularly sensitive to pathology or biological ageing.

The age thresholds after which dual-task performance began to decline with advancing age were consistent with previous neuroimaging evidence, which showed measurable age-related structural or functional deterioration within cognitive brain networks after the age of 55 years.^19–21^ Our results suggest that both processing speed and working memory account for a substantial portion of the observed effect of age on dual-task gait performance. Thus, in adults without any overt neurological or psychiatric disease, the regulation of dual-task gait might share the same underlying neural circuits with that of attention or working memory, or both.^22^ Therefore, age-related alterations in the structural or functional characteristics of this circuit might (at least partly) underlie decrements in dual-task gait performance that manifest after age 54 years.

Although regression analyses showed an effect of age on dual-task gait performance after the age of 54 years and 57 years, residuals exist between an individual’s predicted and measured dual-task performance (figure 1). This unexplained variance suggests that chronological age does not fully capture the totality of the complex locomotor control system that gives rise to the ability to maintain gait performance while engaging in an unrelated cognitive task. This mismatch between chronological and biological age has been observed in numerous human physiological systems. Steffener and colleagues reported that, at the same chronological age, participants with higher education had a younger brain age (ie, higher grey matter volume).^23^ Studies have also reported that markers of biological age might more appropriately reflect the level of a system’s functionality than chronological age and are thus more predictive of later-life health and mortality.^24,25^ Future prospective studies are therefore needed to examine whether the magnitude of observed residuals between actual and age-predicted performance could be linked to individual resilience to ageing and age-related conditions and thus to personal risk of health issues in later life.

Several prospective studies in adults aged 65 years or older have, in fact, linked baseline dual-task gait performance to the risk of falls and cognitive decline.^4,26^ The observation that increased DTCs to gait might manifest well before the age of 65 years suggests that this metric might serve as a clinically meaningful, early indicator of accelerated ageing, otherwise presymptomatic neurodegenerative conditions, or both. Although the value of monitoring dual-task performance in middle age has not been extensively studied, such an approach might enable recommendation of preventive interventions, including those that have been reported to mitigate DTCs to gait (eg, dual-task training), or even non-invasive brain stimulation.^27,28^

At the initiation of the BBHI study, no participants had age-related neurological diseases, cognitive decline, or neuromuscular disorders. How these or other conditions might influence the observed effects of age and cognition in middle age on dual-task gait performance is unknown. Moreover, we focused only on stride time and stride time variability as markers of locomotor control. Although these metrics are well established and are highly associated with other gait metrics (eg, speed) in healthy individuals,^29^ other gait metrics might not be similarly influenced by the independent variables (eg, cognitive processing speed and working memory) of this study.^30^ Many underlying neurophysiological elements pertaining to gait regulation were not measured (eg, peripheral sensorimotor function). The observations here were based on the cross-sectional secondary analyses, which might induce the issues of reverse causality and incidence–prevalence bias. Nevertheless, to our knowledge, this study provides first-of-its-kind evidence that dual-task gait performance starts diminishing up to a decade before traditionally defined older age (ie, aged ≥65 years), and that such declines are associated with interindividual variance in cognitive function. Longitudinal, well powered studies are warranted to establish the causal relationships between changes over time in dual-task gait performance and neuropathology in the brain and the association between earlier-than-expected increases in DTC and adverse health outcomes in later life.

## Supplementary Material

1

## Figures and Tables

**Table 1:** Participant characteristics and dual-task gait performance

	Full BBHI sample (n=996)	Study population (n=640)	Younger group (n=331 [52%])	Older group (n=309 [48%])
Age, years	53 (46–58)	53 (47–58)	47 (44–50)	58 (56–62)
Sex				
Women	470 (47%)	298 (47%)	156 (47%)	142 (46%)
Men	526 (53%)	342 (53%)	175 (53%)	167 (54%)
Race or ethnicity				
White	823 (83%)	547 (85%)	290 (88%)	257 (83%)
Black	2 (<1%)	0	0	0
Asian	3 (<1%)	2 (<1%)	2 (1%)	0
Native American	5 (1%)	4 (1%)	1 (<1%)	3 (1%)
Malay	3 (<1%)	0	0	0
Other	62 (6%)	34 (5%)	16 (5%)	18 (6%)
No response	98 (10%)	53 (8%)	22 (7%)	31 (10%)
Time spent in education, years	17 (16–19)	17 (15–19)	17 (15–19)	17 (14–19)
BMI	25·5 (22·4–28·3)	25·1 (22·8–28·2)	24 (21·8–27·2)	26 (23·4–28·8)
MMSE score	30 (30–30)	30 (30–30)	30 (30–30)	30 (30–30)
SPPB score	12 (12–12)	12 (12–12)	12 (12–12)	12 (12–12)
Stride time				
Single task, s	NA	1·05 (1·01–1·12)	1·05 (1·01–1·12)	1·05 (1·00–1·11)
Dual task, s	NA	1·05 (1·04–1·15)	1·10 (1·04–1·17)	1·10 (1·04–1·18)
DTC, %	NA	3·7% (1·4–7·0)	4·0% (1·2–7·1)	4·2% (1·5–7·8)
Stride time variability				
Single task	NA	0·02 (0·02–0·03)	0·02 (0·02–0·03)	0·02 (0·02–0·03)
Dual task	NA	0·02 (0·02–0·03)	0·02 (0·02–0·03)	0·02 (0·02–0·04)
DTC, %	NA	17·7% (–6·9 to 52·0)	15·0% (–11·6 to 50·4)	23·0% (–2·2 to 55·8)
Correct counting rate in dual-task walking, %	NA	100% (91–100)	100% (92–100)	100% (89–100)

Data are median (IQR) or n (%). The age threshold for groups was based on the results of our primary outcomes of gait (ie, DTC to gait). Therefore, the younger group consisted of participants aged <54 years and older group consisted of participants aged ≥54 years. Stride time variability was assessed by use of the coefficient of the stride time. BBHI=Barcelona Brain Health Initiative study. DTC=dual-task cost. MMSE=Mini Mental State Examination. NA=not applicable. SPPB=Short Physical Performance Battery.

**Table 2:** Associations between cognitive domains and gait characteristics within the older group (ie, aged ≥54 years) as assessed by multivariable models

	Dual-task stride time variability		Dual-task cost to stride time		Dual-task cost to stride time variability	
	Standardised β (95% CI)	p value	Standardised β (95% CI)	p value	Standardised β (95% CI)	p value
Processing speed	−0·27 (−0·38 to −0·18)	0·011	−0·20 (−0·38 to −0·12)	p=0·021	−0·22 (−0·29 to −0·04)	0·013
Working memory	−0·18 (−0·25 to −0·03)	0·032	−0·18 (−0·32 to −0·07)	p=0·020	−0·20 (−0·33 to −0·09)	0·022
Episodic memory	−0·08 (−0·17 to 0·12)	0·19	−0·06 (−0·09 to 0·03)	p=0·41	−0·01 (−0·06 to 0·20)	0·22
Flexibility	−0·10 (−0·19 to 0·07)	0·16	−0·09 (−0·13 to 0·04)	p=0·41	−0·07 (−0·14 to 0·08)	0·16
Reasoning	−0·11 (−0·21 to 0·03)	0·15	−0·14 (−0·23 to 0·08)	p=0·078	−0·05 (−0·11 to 0·05)	0·17

## Data Availability

The data that support the findings of this study are available from the Guttmann University Institute, Universitat Autònoma de Barcelona, Barcelona, Spain. All deidentified raw data of each participant in the Barcelona Brain Health Initiative study are accessible on reasonable request to David Bartrés-Faz (dbartres@ub.edu) following appropriate research ethical approvals and with permission of the authors.

## Research in context

### Evidence before this study

We searched PubMed, Web of Science, and Google Scholar for studies in English published from database inception until Oct 1, 2022. We aimed to identify known associations between dual-task gait performance, age, and cognitive function in middle-aged adults. We used the combination of search terms: (“dual task gait” OR “dual task walking”) AND (“middle age” OR “mid-aged” OR “middle-aged”) AND (“cognition” OR “cognitive function”). To date, most published studies have cross-sectionally compared gait or dual-task gait performance, or both, between younger, older, and middle-aged groups.

We found no published studies that specifically examined the association between age and dual-task gait performance within the middle-aged population or the contribution of cognitive function to observed age-related changes in dual-task gait performance within this segment of the population.

### Added value of this study

To our knowledge, this is the first study to characterise the associations between age, dual-task gait performance, and cognitive function in healthy middle-aged adults. Our findings indicate that, although gait performance under usual conditions (ie, walking without simultaneously performing other cognitive tasks) does not change as a function of age in this segment of the population, dual-task gait performance starts to decline in the middle of the sixth decade of life.

These results suggest that dual-task gait performance might be an early indicator of accelerated brain ageing or otherwise presymptomatic neurodegenerative conditions.

### Implications of all the available evidence

Poor dual-task gait performance has been linked to risk of major cognitive impairment, falls, and brain health alterations in older adults (ie, aged ≥65 years). Our results further suggest that dual-task walking is an important functional ability that should be routinely monitored starting in middle age.

## References

[1] Yogev-Seligmann G, Hausdorff JM, Giladi N. The role of executive function and attention in gait. Mov Disord 2008; 23: 329–42. 18058946 10.1002/mds.21720 PMC2535903

[2] Hausdorff JM, Schweiger A, Herman T, Yogev-Seligmann G, Giladi N. Dual-task decrements in gait: contributing factors among healthy older adults. J Gerontol A Biol Sci Med Sci 2008; 63: 1335–43. 19126846 10.1093/gerona/63.12.1335 PMC3181497

[3] Haworth JM. Gait, aging and dementia. Rev Clin Gerontol 2008; 18: 39–52.

[4] Montero-Odasso MM, Sarquis-Adamson Y, Speechley M, Association of dual-task gait with incident dementia in mild cognitive impairment: results from the Gait and Brain Study. JAMA Neurol 2017; 74: 857–65. 28505243 10.1001/jamaneurol.2017.0643 PMC5710533

[5] Pieruccini-Faria F, Black SE, Masellis M, Gait variability across neurodegenerative and cognitive disorders: results from the Canadian Consortium of Neurodegeneration in Aging (CCNA) and the Gait and Brain Study. Alzheimers Dement 2021; 17: 1317–28. 33590967 10.1002/alz.12298 PMC8451764

[6] MacAulay RK, Wagner MT, Szeles D, Milano NJ. Improving sensitivity to detect mild cognitive impairment: cognitive load dual-task gait speed assessment. J Int Neuropsychol Soc 2017; 23: 493–501. 28413999 10.1017/S1355617717000261

[7] Brustio PR, Magistro D, Zecca M, Rabaglietti E, Liubicich ME. Age-related decrements in dual-task performance: comparison of different mobility and cognitive tasks. A cross sectional study. PLoS One 2017; 12: e0181698. 28732080 10.1371/journal.pone.0181698 PMC5521845

[8] Hollman JH, Kovash FM, Kubik JJ, Linbo RA. Age-related differences in spatiotemporal markers of gait stability during dual task walking. Gait Posture 2007; 26: 113–19. 16959488 10.1016/j.gaitpost.2006.08.005

[9] Kivipelto M, Ngandu T, Laatikainen T, Winblad B, Soininen H, Tuomilehto J. Risk score for the prediction of dementia risk in 20 years among middle aged people: a longitudinal, population-based study. Lancet Neurol 2006; 5: 735–41. 16914401 10.1016/S1474-4422(06)70537-3

[10] Cattaneo G, Bartrés-Faz D, Morris TP, The Barcelona Brain Health Initiative: a cohort study to define and promote determinants of brain health. Front Aging Neurosci 2018; 10: 321. 30405394 10.3389/fnagi.2018.00321 PMC6204574

[11] Mitchell AJ. A meta-analysis of the accuracy of the Mini-Mental State Examination in the detection of dementia and mild cognitive impairment. J Psychiatr Res 2009; 43: 411–31. 18579155 10.1016/j.jpsychires.2008.04.014

[12] Manor B, Yu W, Zhu H, Smartphone app–based assessment of gait during normal and dual-task walking: demonstration of validity and reliability. JMIR Mhealth Uhealth 2018; 6: e36. 29382625 10.2196/mhealth.8815 PMC5811655

[13] Su D, Liu Z, Jiang X, Simple smartphone-based assessment of gait characteristics in Parkinson disease: validation study. JMIR Mhealth Uhealth 2021; 9: e25451. 33605894 10.2196/25451 PMC7935653

[14] Cattaneo G, Solana-Sánchez J, Abellaneda-Pérez K, Sense of coherence mediates the relationship between cognitive reserve and cognition in middle-aged adults. Front Psychol 2022; 13: 835415. 35418913 10.3389/fpsyg.2022.835415 PMC8996461

[15] Tennant PWG, Murray EJ, Arnold KF, Use of directed acyclic graphs (DAGs) to identify confounders in applied health research: review and recommendations. Int J Epidemiol 2021; 50: 620–32. 33330936 10.1093/ije/dyaa213 PMC8128477

[16] Cleveland WS, Devlin SJ. Locally weighted regression: an approach to regression analysis by local fitting. J Am Stat Assoc 1988; 83: 596–610.

[17] Leone C, Feys P, Moumdjian L, D’Amico E, Zappia M, Patti F. Cognitive-motor dual-task interference: a systematic review of neural correlates. Neurosci Biobehav Rev 2017; 75: 348–60. 28104413 10.1016/j.neubiorev.2017.01.010

[18] Bayot M, Dujardin K, Tard C, The interaction between cognition and motor control: a theoretical framework for dual-task interference effects on posture, gait initiation, gait and turning. Neurophysiol Clin 2018; 48: 361–75. 30487064 10.1016/j.neucli.2018.10.003

[19] Fjell AM, Walhovd KB. Structural brain changes in aging: courses, causes and cognitive consequences. Rev Neurosci 2010; 21: 187–221. 20879692 10.1515/revneuro.2010.21.3.187

[20] Nóbrega-Sousa P, Gobbi LTB, Orcioli-Silva D, Conceição NRD, Beretta VS, Vitório R. Prefrontal cortex activity during walking: effects of aging and associations with gait and executive function. Neurorehabil Neural Repair 2020; 34: 915–24. 32865134 10.1177/1545968320953824

[21] Hoang I, Paire-Ficout L, Derollepot R, Perrey S, Devos H, Ranchet M. Increased prefrontal activity during usual walking in aging. Int J Psychophysiol 2022; 174: 9–16. 35093479 10.1016/j.ijpsycho.2022.01.011

[22] Ghanavati T, Smitt MS, Lord SR, Deep white matter hyperintensities, microstructural integrity and dual task walking in older people. Brain Imaging Behav 2018; 12: 1488–96. 29297156 10.1007/s11682-017-9787-7

[23] Steffener J, Habeck C, O’Shea D, Razlighi Q, Bherer L, Stern Y. Differences between chronological and brain age are related to education and self-reported physical activity. Neurobiol Aging 2016; 40: 138–44. 26973113 10.1016/j.neurobiolaging.2016.01.014 PMC4792330

[24] Levine ME. Modeling the rate of senescence: can estimated biological age predict mortality more accurately than chronological age? J Gerontol A Biol Sci Med Sci 2013; 68: 667–74. 23213031 10.1093/gerona/gls233 PMC3660119

[25] Belsky DW, Caspi A, Houts R, Quantification of biological aging in young adults. Proc Natl Acad Sci USA 2015; 112: E4104–10. 26150497 10.1073/pnas.1506264112 PMC4522793

[26] Muir-Hunter SW, Wittwer JE. Dual-task testing to predict falls in community-dwelling older adults: a systematic review. Physiotherapy 2016; 102: 29–40. 26390824 10.1016/j.physio.2015.04.011

[27] Bherer L, Kramer AF, Peterson MS, Colcombe S, Erickson K, Becic E. Transfer effects in task-set cost and dual-task cost after dual-task training in older and younger adults: further evidence for cognitive plasticity in attentional control in late adulthood. Exp Aging Res 2008; 34: 188–219. 18568979 10.1080/03610730802070068 PMC2845439

[28] Schneider N, Dagan M, Katz R, Combining transcranial direct current stimulation with a motor-cognitive task: the impact on dual-task walking costs in older adults. J Neuroeng Rehabil 2021; 18: 23. 33526043 10.1186/s12984-021-00826-2 PMC7852224

[29] Beauchet O, Annweiler C, Lecordroch Y, Walking speed-related changes in stride time variability: effects of decreased speed. J Neuroeng Rehabil 2009; 6: 32. 19656364 10.1186/1743-0003-6-32 PMC2731039

[30] Lo OY, Halko MA, Zhou J, Harrison R, Lipsitz LA, Manor B. Gait speed and gait variability are associated with different functional brain networks. Front Aging Neurosci 2017; 9: 390. 29249961 10.3389/fnagi.2017.00390 PMC5715372

