# Supplementary material for: The age-related contribution of cognitive function to dual-task gait in middle-aged adults in Spain: observations from a population-based study
Source: Lancet Healthy Longev. Author manuscript; Available in PMC 2023 Mar 8. (PMC9992865; doi:10.1016/S2666-7568(23)00009-0)
Supplement: 1 [file NIHMS1879284-supplement-1.pdf]

THE LANCET
Healthy Longevity

Supplementary appendix

This appendix formed part of the original submission and has been peer reviewed. We post it as supplied by the authors.

Supplement to: Zhou J, Cattaneo G, Yu W, et al. The age-related contribution of cognitive function to dual-task gait in middle-aged adults in Spain: observations from a population-based study. *Lancet Healthy Longev* 2023; **4:** e98–106.

Supplementary Materials

Study sample size

This secondary analysis of the BBHI study included all participants that completed assessments of both dual task gait and cognitive function. Power calculations indicated that an evaluable sample size of 640 resulted in a minimum detectable effect size of 0.01 in regression models including up to five predictors (alpha = 0.05; statistical power (1-beta) = 0.80).

Clinical assessment

The clinical assessment was conducted by a physician and/or a licensed physical therapist and included a standard personal and family-based medical history, review of prescribed medications, self-report of drugs and substance consumption, cardio-vascular risk factors, anthropometrics, hand dominance, and the administration of the Short Physical Performance Battery (SPPB).

Gait assessment

Participants were asked to wear comfortable shoes and pants. The gait assessment was completed within a hallway along a 26-meter course free of doorways, windows, foot traffic, or other distraction. An iOS gait assessment application (App) was used to record gait-related data. The same smartphone was used for all gait assessments across all participants. The App was developed and validated by Manor et al[11]. Briefly, the App provides a combination of video, text, and finally, voice instructions to guide the participant through a series of walking trials with the smartphone placed in their pants front pocket. In this study, the research staff member was present during all assessments to provide additional explanation of procedures and supervise the participant. Each assessment consisted of one 45-second trial of normal walking (i.e., single-task) followed by one 45-second trial of walking while performing a cognitive serial-subtraction task (i.e., dual-task) as designed in the App. This specific cognitive task was chosen because it is the one most commonly implemented in older adults, often disrupts gait in even healthy younger adults, and produces dual-task costs that are sensitive to aging and cognitive decline[4]. At the beginning of dual-task walking trial, the App provided a random three-digit number between 100 and 999 and the participant then counted backwards by three from that number throughout the trial. The participant was encouraged to continue counting backwards by 3's for the entire trial, even if they believed they made a mistake. Participants were instructed to walk at their preferred speed along a hallway that was approximately 20 meters long, make a 180 degree of turn at the end of the hallway, and continue to walk until instructed to stop by the App. Smartphone inertial measurement unit (IMU) data and serial subtraction performance (i.e., percentage of correct responses) were recorded during each trial.

Analysis of gait metrics

As described previously[12,13], the IMU contained within the smartphone captured 3-dimensional acceleration and angular velocity time series of each walking trial at a sampling frequency of 100 Hz. The App automatically uploaded these signals to a cloud-based server for offline analysis. Raw time series were transformed from the device coordinate systems to the global coordinate system by using the quaternion rotation matrix. By doing so, the transformed Z-axis was thus aligned with the gravitational axis of the earth regardless of the orientation of the phone placed in the pocket. Each rotated times-series was then filtered using a low-pass Butterworth filter with the cut-off frequency of 3 Hz. As previously described, periods of turning while walking along the hallway course were identified by determining relatively prolonged periods of non-zero crossings within the filtered angular velocity time-series about the vertical axis with respect to gravity[4]. Notably, relatively rapid 180 degree turns result in an area under the curve between 2 consecutive zero crossings to be equal to approximately $\pi/2$. For this study, this data driven approach was further verified by comparing to staff-recorded timing of turns relative to the start of each trial. Once a turn was identified and verified, the entire portion of the trial between the prolonged non-zero crossings was removed. We then identified each heel strike of the phone-side leg from the Z-axis acceleration data using a method validated against goal-standard instrumentation for gait assessment[4]. These heel strike data were used to compute the primary outcomes of stride time and stride time variability from each trial. To help ensure that these outcomes were derived from periods of relatively straight, steady-state walking, we additionally removed the first two and last two identified strides of each trial, as well as one validated stride on either side of the omitted turn. All participants successfully completed the walking trials and the number of strides used to compute primary outcomes ranged from 20-43 with an average of 33.

The construction of composite score of cognitive performance

The neuropsychological battery consisted of the following tests: general/fluid intelligence and reasoning (Wechsler Adult Intelligence Scale (WAIS-IV) Matrices, Block design); visuo-spatial abilities and memory (Corsi block test); visuo-spatial searching, attention and visuo-motor speed (Cancelation test WAIS-IV, digit symbol substitution, and Trial making test-A(TMT-A)); cognitive flexibility (Trial making test B and B-A, TMT-B-A); working memory (Digit span forward, Digit backward and Letter number sequencing); and episodic memory (Rey Auditory Verbal Learning Test, RAVLT). To construct the composite score, scores on individual tests were transformed into z-scores and entered into factor analysis (FA) with Oblimin rotation which allows possible correlation between latent factors.

Based on the sample size, the acceptable level of factor loading was set at 0.30. Bartlett's test revealed a significant relationship between the factors ($p < 0.015$) and The Kaiser–Meyer–Olkin test confirmed that the data were acceptable for factor analysis (KMO= 0.62).

PCA indicates the presence of five principal components, explaining 68.30% of the total variance of cognitive scores. The first factor included the cancellation test (0.78), the TMT-A (0.76) and the digit symbol test (0.64), likely reflecting visual searching, processing speed and attentional components. The second component comprised all three measures of the RAVLT (immediate recall = 0.88, delayed recall = 0.88, recognition = 0.85) converging in a verbal episodic memory domain. The third component contained the digit forward (0.83), digit backward (0.72) and letter-number sequencing (0.63), reflecting a working memory domain. Cognitive flexibility and set-shifting abilities were reflected in the fourth component which included the TMT-B (0.99) and the TMT-B-A (0.91). Finally, a visuospatial reasoning and problem-solving domain was found in the fifth component comprising WAIS-IV matrix reasoning (0.83), block design (0.72) and Corsi cubes (0.34). The composite scores of those cognitive domains were then calculated as the weighted sum of the z-scores from each test per the results of the PCA analysis (appendix p 4). Based on these five factors, we also constructed the global composite cognitive score.

Figure S1. The causal directed acyclic graph (cDAG) informing regression and SEM models examining the relationships between age, dual-task cost to gait, and cognitive function. This cDAG was framed based upon the primary hypotheses of this work. The primary outcome in both regression and SEM models was dual task ‘cost’ to gait, which is determined by calculating the relative change in gait performance from single- to dual-task conditions. This outcome thus controls for many of the peripheral sensorimotor elements of the locomotor control system (as these elements are the same in both walking conditions), and therefore reflects primarily the functionality of the higher-level brain functions that enable dual tasking. In regression analyses, cognitive function was the predictor. There is strong evidence indicating that biological age affects numerous underlying neurophysiological systems subserving both cognitive function and the dual-task cost to gait (e.g., visual acuity, cardiorespiratory function, metabolism, etc.). Biological age was therefore designated as a latent variable that captures these unmeasured confounders and included as a covariate in regression models. Within SEM analyses, we anticipated that the observed total association between age (predictor) and dual-task cost to gait may be mediated by cognitive function (the path in light blue). Based upon previous literature exploring the relationships between age, cognitive function and dual-task cost to gait, and considering that the cohort was relatively healthy (e.g., no medication use, no psychiatric or mood conditions, no physical disabilities, etc.), we designated BMI and sex as measured confounders (grey circles) and included them as covariates of both regression and SEM models.

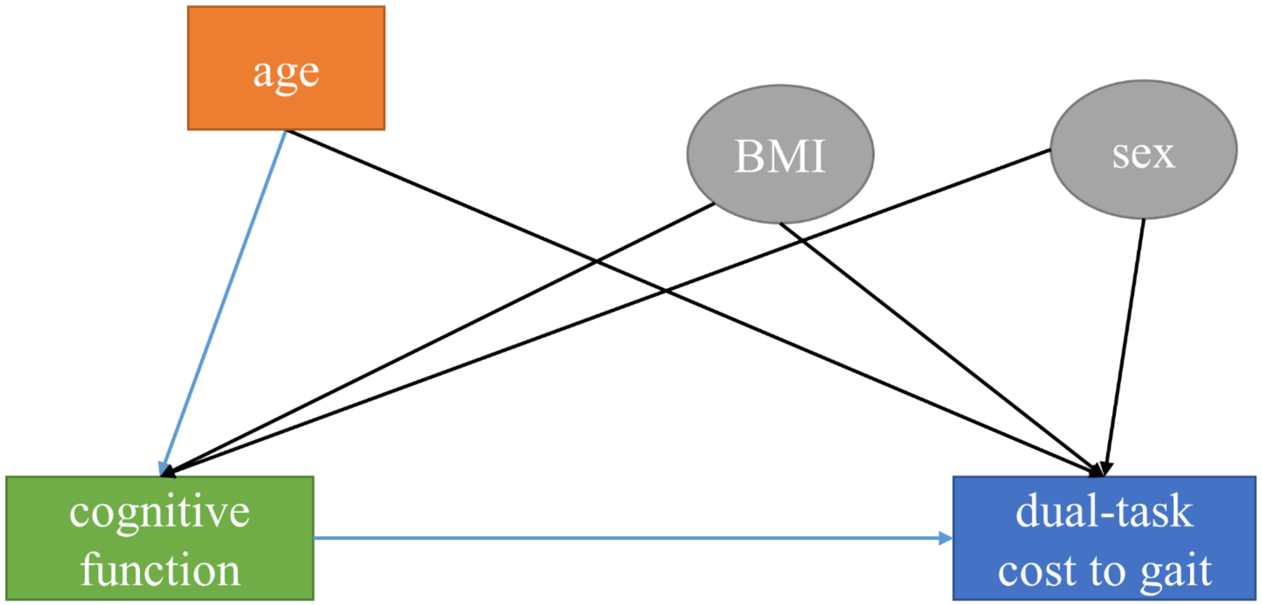

Supplementary Table 1. Results of the principal component analysis (PCA) on cognitive scores.

Neuropsychological test	Factor 1	Factor 2	Factor 3	Factor 4	Factor 5
Cancellation	0.777				
Trial making test A	-0.760				
Digit symbol	0.638				
RAVLT delayed recall		0.884			
RAVLT immediate recall		0.880			
RAVLT recognition		0.849			
Digit span forward			0.833		
Digit span backward			0.716		
Number-Letter sequencing			0.628		
Trial making test B				0.997	
Trial making test B-A				0.914	
WAIS-IV Matrix					0.826
Block design					0.724
Corsi tap cubes					0.343

Columns represent the structure of the model and the 5 different latent variables. Individual cell numbers represent the factorial loading coefficient.

Supplmentary Table 2. Cogntive test performance.

Mean (S.D.) (range, median, interquartile range)	Study population (n=640)	Younger group (age<54 years) (n=331)	Older group (age>=54 years) (n=309)	p value
TMT_(B-A) (s)	50 (22.8) (11-217, 47, 30)	47.1 (19.1) (11-127, 44, 24)	56.2 (28.3) (11-217, 50, 33)	<0.001
Digit symbol	78.5 (13.2) (42-114, 78, 19)	80.9 (12.8) (47-114, 81, 17)	75.2 (13.1) (42-111, 74, 20)	<0.001
Digit forward	6.23 (1.19) (4-9, 6, 2)	6.1 (1.2) (4-9, 6, 2)	6.3 (1.2) (4-9, 6, 2)	0.21
Digit backward	4.95 (1.09) (2-9, 5, 2)	5 (1.1 (2-8, 5, 2)	4.9 (1.1) (2-9, 5, 2)	0.17
Cancellation	41.99 (8.29) (21-66, 41, 11)	42.9 (8.5) (12-66, 43, 12)	40.6 (7.8) (21-60, 40, 11)	<0.001
WAIS-IV	20.1 (3.3) (6-26, 21, 5)	20.8 (2.9) (9-26, 21, 3.5)	19.3 (3.5) (6-26, 20, 5)	<0.001
Corsi_cubes	6.52 (0.94) (4-9, 7, 1)	6.6 (8.5) (4-9, 7, 1)	6.3 (0.9) (4-9, 7, 1)	<0.001
Block_design	46.53 (10.13) (20-65, 48, 16)	48.4 (9.6) (20-65, 50, 13)	44.4 (10.4) (24-63, 45, 18)	<0.001
Letter_number_sequence	5.75 (1.06) (3-8, 6, 1)	5.8 (1.1) (4-8, 6, 2)	5.6 (1) (3-8, 6, 1)	0.021
RAVLT_immediate	51.78 (8.64) (0-71, 52, 12)	53.7 (8.5) (0-71, 54, 10)	49.1 (8.5) (25-69, 49, 12)	<0.001
RAVLT_delayed	11.27 (2.71) (2-15, 12, 4)	22.8 (2.5) (2-15, 12, 4)	10.6 (2.8) (4-15, 11, 5)	<0.001
RAVLT_recognizing	14.36 (1.18) (8-15, 15, 1)	14.5 (0.9) (8-15, 15, 1)	14.2 (1.4) (8-15, 15, 1)	<0.001
Composite scores				
Global cognitive function	0.1 (8.1) (-34.4-21.5, 0.5, 11.4)	2.4 (7.3) (-23.9-19.3, 2.6, 9.9)	-2.6 (8.6) (-34.4-21.5, -2.2, 11.8)	<0.001
processing speed	0.007 (2.3) (-7.2-6.9, -0, 3.1)	0.59 (2.2) (-6-6.9, 0.6, 2.9)	-0.65 (2.2) (-7.2-6.1, -0.6, 3.1)	<0.001
working memory	0.003 (2.7) (-6.8-8.1, -17, 3.7)	0.25 (2.7) (-6.8-7.8, -0.14, 3.9)	-0.27 (2.6) (-5.9-8.1, -0.27, 3.7)	0.013

episodic memory	0.03 (1.8) (-11.1-4.1, -0.5, 3.3)	0.59 (2.3) (-7.4-4.1, 1.1, 2.9)	-0.65 (2.7) (-11.1-3.9, -0.19, 3.4)	<0.001
flexibility	0.008 (1.7) (-9.6-3.5, 0.2, 2.3)	0.42 (1.5) (-6.4-3.5, 0.7, 1.9)	-0.46 (2.3) (-9.6-2.9, -0.02, 2.5)	<0.001
reasoning	0.12 (1.6) (--4.8-3.4, 0.17, 2.4)	0.4 (1.5) (-4.8-3.4, 0.7, 1.9)	-0.43 (1.7) (-6.1-3.2, -0.18, 2.6)	<0.001

Table S3. The results of covariates in the regression models examining the relationships between global cognitive function and dual-task gait performance within the designated older group (aged ≥54 years).

β (95%CI), p value	dual-task cost to stride time	dual-task cost to stride time variability	stride time variability within the dual-task condition
age	β=0.05 (95%CI: -0.06-0.12), p=0.13	β=0.08 (95%CI: -0.04-0.13), p=0.25	β=0.04 (95%CI: -0.03-0.11), p=0.15
sex	β=0.01 (95%CI: -0.08-0.05), p=0.34	β=-0.02 (95%CI: -0.05-0.06), p=0.18	β=0.01 (95%CI: -0.05-0.06), p=0.34
Body mass index (BMI)	β=0.03 (95%CI: -0.04-0.07), p=0.83	β=0.04 (95%CI: -0.06-0.08), p=0.44	β=0.02 (95%CI: -0.09-0.08), p=0.82

Table S4. The results of covariates in the multivariable regression models examining the relationships between cognitive domains and dual-task gait performance within older group (age≥54 years).

β (95%CI), p value	dual-task cost to stride time	dual-task cost to stride time variability	stride time variability within the dual-task condition
age	β=0.06 (95%CI: -0.07-0.11), p=0.14	β=0.05 (95%CI: -0.11-0.1), p=0.17	β=0.02 (95%CI: -0.07-0.17), p=0.83
sex	β=0.04 (95%CI: -0.08-0.11), p=0.24	β=0.01 (95%CI: -0.06-0.09), p=0.98	β=0.03 (95%CI: -0.07-0.11), p=0.18
body mass index (BMI)	β=0.02 (95%CI: -0.08-0.09), p=0.53	β=0.01 (95%CI: -0.05-0.07), p=0.97	β=0.04 (95%CI: -0.07-0.16), p=0.12
